# Long-term adherence to a wearable for continuous behavioural activity measuring in the SafeHeart implantable cardioverter defibrillator population

**DOI:** 10.1093/ehjdh/ztae055

**Published:** 2024-08-01

**Authors:** Diana My Frodi, Maarten Z H Kolk, Joss Langford, Reinoud Knops, Hanno L Tan, Tariq Osman Andersen, Peter Karl Jacobsen, Niels Risum, Jesper Hastrup Svendsen, Fleur V Y Tjong, Søren Zöga Diederichsen

**Affiliations:** Department of Cardiology, Copenhagen University Hospital—Rigshospitalet, Inge Lehmanns Vej 7, DK-2100 Copenhagen, Denmark; Department of Clinical and Experimental Cardiology, Amsterdam UMC, University of Amsterdam, Meibergdreef 9, 1105 AZ Amsterdam, The Netherlands; Activinsights Ltd, 6 Nene Road, Bicton Industrial Park, Kimbolton, Cambridgeshire, PE28 0LF, UK; College of Life and Environmental Sciences, University of Exeter, St Luke's Campus, Heavitree Road, Exeter, EX1 2LU, UK; Department of Clinical and Experimental Cardiology, Amsterdam UMC, University of Amsterdam, Meibergdreef 9, 1105 AZ Amsterdam, The Netherlands; Department of Clinical and Experimental Cardiology, Amsterdam UMC, University of Amsterdam, Meibergdreef 9, 1105 AZ Amsterdam, The Netherlands; Netherlands Heart Institute, Moreelsepark 1, 3511 EP Utrecht, The Netherlands; Department of Computer Science, University of Copenhagen, Universitetsparken 1, DK-2100 Copenhagen, Denmark; Department of Cardiology, Copenhagen University Hospital—Rigshospitalet, Inge Lehmanns Vej 7, DK-2100 Copenhagen, Denmark; Department of Cardiology, Copenhagen University Hospital—Rigshospitalet, Inge Lehmanns Vej 7, DK-2100 Copenhagen, Denmark; Department of Cardiology, Copenhagen University Hospital—Rigshospitalet, Inge Lehmanns Vej 7, DK-2100 Copenhagen, Denmark; Department of Clinical Medicine, Faculty of Health and Medical Sciences, University of Copenhagen, Blegdamsvej 3B, DK-2100 Copenhagen, Denmark; Department of Clinical and Experimental Cardiology, Amsterdam UMC, University of Amsterdam, Meibergdreef 9, 1105 AZ Amsterdam, The Netherlands; Department of Cardiology, Copenhagen University Hospital—Rigshospitalet, Inge Lehmanns Vej 7, DK-2100 Copenhagen, Denmark

**Keywords:** Wearable, Implantable cardioverter defibrillator, Adherence, Prediction, Activity tracker, Long term

## Abstract

**Aims:**

Wearable health technologies are increasingly popular. Yet, wearable monitoring only works when devices are worn as intended, and adherence reporting lacks standardization. In this study, we aimed to explore the long-term adherence to a wrist-worn activity tracker in the prospective SafeHeart study and identify patient characteristics associated with adherence.

**Methods and results:**

This study enrolled 303 participants, instructed to wear a wrist-worn accelerometer day and night for 6 months. Long-term adherence was defined as valid days (≥22 h of wear time) divided by expected days, and daily adherence as mean hours of wear time per 24 h period. Optimal, moderate, and low long-term and daily adherence groups were defined as long-term adherence above or below 95 and 75% and daily adherence above or below 90 and 75%. Regression models were used to identify patient characteristics associated with long-term adherence. In total, 296 participants [median age 64 years; interquartile range (IQR) 57–72; 19% female] were found eligible, yielding 44 003 days for analysis. The median long-term adherence was 88.2% (IQR 74.6–96.5%). A total of 83 (28%), 127 (42.9%), and 86 (29.1%) participants had optimal, moderate, and low long-term adherence, and 163 (55.1%), 87 (29.4%), and 46 (15.5%) had optimal, moderate, and low daily adherence, respectively. Age and smoking habits differed significantly between adherence levels, and increasing changeover intervals improved the degree of long-term adherence.

**Conclusion:**

Long-term adherence to a wearable activity tracker was 88.2% over a 6-month period. Older age and longer changeover interval were positively associated with long-term adherence. This serves as a benchmark for future studies that rely on wearable devices.

**Trial registration number:**

The National Trial Registration number: NL9218 (https://onderzoekmetmensen.nl/).

## Graphical Abstract

**Graphical Abstract:**
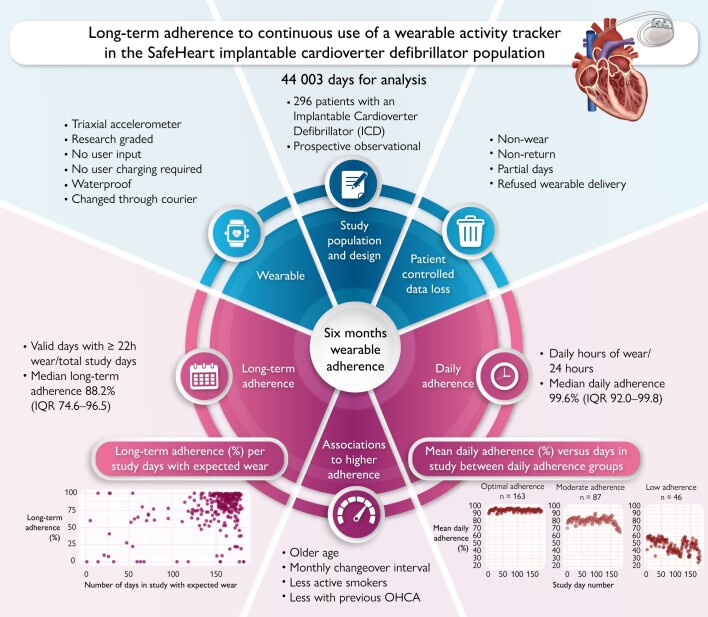

## Introduction

Wearable sensors have gained popularity over the past decade as a means of continuous and objective data collection.^1,2^ With increased granularity in data, fluctuations and trends over time can be captured with the potential of strengthening preventive strategies in follow-up care.^3^ For these devices to uphold their efficacy, it is pivotal that patients sustain their use over time.^4,5^ The reliability of data collected through wearable devices depends not only on adequate adherence, but importantly also on the technological capabilities and user requirements of the device, the digital health literacy of participants, and the mechanisms of data return - all of which, in turn, interact with the level of adherence.^4,6^

The knowledge on adherence to wearables remains limited and uniformity of how to define or subcategorize adherence is lacking, making comparability and generalizability across studies challenging.^2^ Furthermore, there is currently a lack of reporting on continuous, long-term adherence to wearables, where the large studies of Fitbit or Apple Watch use for atrial fibrillation (AF) detection have not reported on adherence to the actual wearable but have focused on the logistics pertaining to the studies [scheduling a study visit after app notification, returning electrocardiogram (ECG) patch after use, etc.].^2,7,8^

Prior studies have investigated adherence over brief and intermittent intervals,^9,10^ and some studies have evaluated patient-reported adherence rather than objective measures (e.g. wearable-measured wear time).^11,12^ In a future of increased monitoring, wearables could prove useful mainly for long-term measurements—both in research and in the clinical setting.

Finally, results from healthy participants are not necessarily applicable to patients with chronic illnesses, for instance patients with an implantable cardioverter defibrillator (ICD), in which the use of wearables may be relevant.^13,14^ Therefore, here, we seek to improve the understanding of long-term adherence to wearable activity trackers and factors associated with adherence during 6 months of expected continuous wear, by analysing data from the prospective SafeHeart study on patients with ICD.^15^ Due to the lack of uniformity in defining adherence, our objective applies to adherence with any basic wearable device across diverse patient populations.

## Methods

### Study design

The prospective, observational SafeHeart study enrolled 303 participants from 2 tertiary centres (Copenhagen University Hospital—Rigshospitalet and Amsterdam University Medical Centre) with an ICD with or without resynchronization therapy (cardiovascular resynchronization therapy defibrillator, CRT-D) between May 2021 and September 2022. In this analysis, the first 6 months of follow-up were included. We report findings according to the STROBE guidelines.^16^ The Institutional Review Board and Regional Ethics Committee of both centres approved the study protocol. The study was registered at the National Trial Registration (NL9218, https://onderzoekmetmensen.nl/) and was carried out in accordance with the Declaration of Helsinki. All participants provided written informed consent prior to participation.

### Study population

Participants were included in the SafeHeart study if they had (i) an ICD implanted with or without resynchronization (CRT-D) <5 years prior to enrolment, (ii) undergone ICD therapy (appropriate/inappropriate) or evidence of ventricular arrhythmia <8 years prior to inclusion, and (iii) were at least 18 years of age. Exclusion criteria included end-stage heart failure, <1 year life expectancy, and serious physical impairment. An exhaustive list of these criteria has been published previously.^15^

### Wearable activity tracker

Devices using accelerometry enable a quantification of daily physical behaviour in a continuous and objective fashion. In this study, participants wore the GENEActiv Original 1.1 triaxial wrist-worn accelerometer (Activinsights Ltd, Cambridgeshire, UK) throughout the first 6 months of study participation (see Supplementary material online, *Figure S1*). The wearable captures high-resolution behavioural measures in the form of body movement and accelerations along three axes, captured at 50 Hz and then 20 Hz. These measures relate to daily physical activity, inactivity, and sleep behaviours. An overview of the behavioural metrics and their definitions has been published previously.^17^ Data were extracted from the device via a USB connection after collection. The raw, sensor-level measurements of acceleration (and near-body-temperature) were processed to create daily summaries, including non-wear time, using the open-source CRAN packages GENEAread and GENEAclassify used in the Activinsights R Markdown Sleep report.^18^ Participants did not receive any information from the wearable activity tracker itself during use, and its use did not require a smartphone. The wrist-worn wearable activity tracker was sent by courier initially biweekly, thereafter monthly, with no further user requirements other than continuous wear day and night. No charging was required on the part of the participant, and removal was required only during the use of a sauna and upon changeover of devices. Participants returned the wearable using local postage services with a pre-paid envelope provided with the next device to be worn. Participants did not receive any encouraging prompts, coaching, or notifications for use, other than indirectly so upon receiving a new wearable at changeovers. All information about use was given to participants upon inclusion and was available in the sheet of instructions sent out together with each wearable. The switch from biweekly to monthly changeovers was implemented to primarily reduce shipping costs within the project budget, but also to reduce the resources required to manage shipping and the total number of wearables needed, as well as to lower patient burden. The switch was done gradually over months to minimize the disruption of ongoing delivery schedules for participants already enrolled. There were no benefits of participation in the SafeHeart study, except the participants’ contribution to future improvements in the treatment of patients with ICD.

### Patient-reported outcome measures at baseline

For this study, two patient-reported outcome measures (PROMs) were collected at baseline, namely the two questionnaires: Kansas City Cardiomyopathy Questionnaire (KCCQ) and EQ5D-5L health-related quality of life questionnaire (EQ5D-5L). From the 23-item KCCQ, 5 health domains were calculated, used as continuous variables for the analyses. The EQ5D-5L rendered two scores that were also presented as continuous variables. The detailed description of the PROMs has been published previously.^15,17^

### Outcomes

The endpoint for this sub-study was the long-term and daily adherence to continuous use of a wrist-worn wearable activity tracker during the first 6 months of the study, as defined below. The endpoints were then explored in terms of their association with patient characteristics, including baseline PROMs.

### Daily and long-term adherence

Long-term adherence was defined as the total number of valid days divided by the total expected days per participant, in line with previous studies.^19–21^ Daily adherence was defined as the number of hours of wear within each 24 h period, from which the mean daily adherence was then derived for each participant and study day. A valid day was defined as 22 h of wear time per 24 h period. This ensured that sleep was measured, while allowing for the device to be taken off for short periods of time, e.g. for device cleaning or for a visit to a sauna. With a low threshold for non-wear each day, we wished to ensure a reliable picture of behaviour across day and night, without having to make assumptions for behaviour in the missing hours. The total expected days for each participant was calculated as the total possible days (*n* = 182) minus non-participant-controlled non-wear where the participant could not wear the device because of it being damaged, incorrectly configurated, non-fitting, and lost or delayed due to logistics or due to systemic loss. Changeover days, defined as days when the participant actively needed to swap devices, were excluded, since participants were expected to change devices upon receiving a new device, causing a systemic data loss inherent to the study protocol. Non-wear was categorized as ‘participant-controlled’ if the non-wear was not attributed to by study logistics. Detailed explanations of the different types of days in the analysis are available in Supplementary material online, *Table S1*. Data return was the number of valid days of data returned by participants after exclusion of days of participant-controlled non-wear and non-participant-controlled non-wear, divided by the theoretical total amount of days in study (182 days × number of participants).

Based on the level of long-term and daily adherence, participants were divided into three groups, namely those with optimal, moderate, and low adherences. A cut-off of 95% or higher defined optimal long-term adherence, whereas a cut-off of 90% defined optimal daily adherence (due to the study predefined valid day of at least 22 h of wear). Moderate and low, long-term and daily adherence were both defined by 75–95% adherence for the moderate group and as 75% or lower for each of the adherences for the low-adherence groups. Thresholds are based on the distribution of long-term adherence, as seen in Supplementary material online, *Figure S2*, as a consensus on adherence cut-off points is yet to be established. Long-term adherence was further calculated for the time periods of month one-two, three-four, and five-six, to evaluate adherence group patterns over time.

### Statistical analysis

Continuous variables were presented as mean ± standard deviation, compared by *t*-test for normally distributed variables, and as median [interquartile range (IQR)], compared by Wilcoxon rank-sum test for non-normally distributed variables. Categorical variables were presented as percentage and frequency, compared by χ^2^ test.

The mean daily adherence per study day was compared among adherence groups through repeated measures analysis of variance using the Greenhouse–Geisser sphericity correction. Age per 10-year increment, sex, ICD vs. CRT-D, AF vs. no AF, and previous vs. no heart failure hospitalizations at baseline were examined for a potential association with long-term adherence level (%) through the use of a multivariable linear regression analysis. The results were reported as the estimated change (including 95% confidence intervals) in long-term adherence per unit increase of the independent variables, when the other variables remained constant. The choice of variables aimed to balance the inclusion of clinically meaningful factors that have been shown to differentiate patients with ICD from one another,^22–25^ while avoiding overfitting or overestimating the result. Lastly, seasonality was measured through mean daily adherence per calendar month from December 2021 to November 2022, where the highest number of participants was enrolled simultaneously. Sensitivity analyses were performed for baseline characteristics and baseline patient-reported outcomes by splitting the population into only 2 groups, those with an adherence of 75% or more and those below. The threshold for statistically significant results was a two-sided *P*-value of ≤0.05. All statistical analyses were performed using R statistical software (version 4.2.2, R Core Team).

## Results

**Figure 1:**
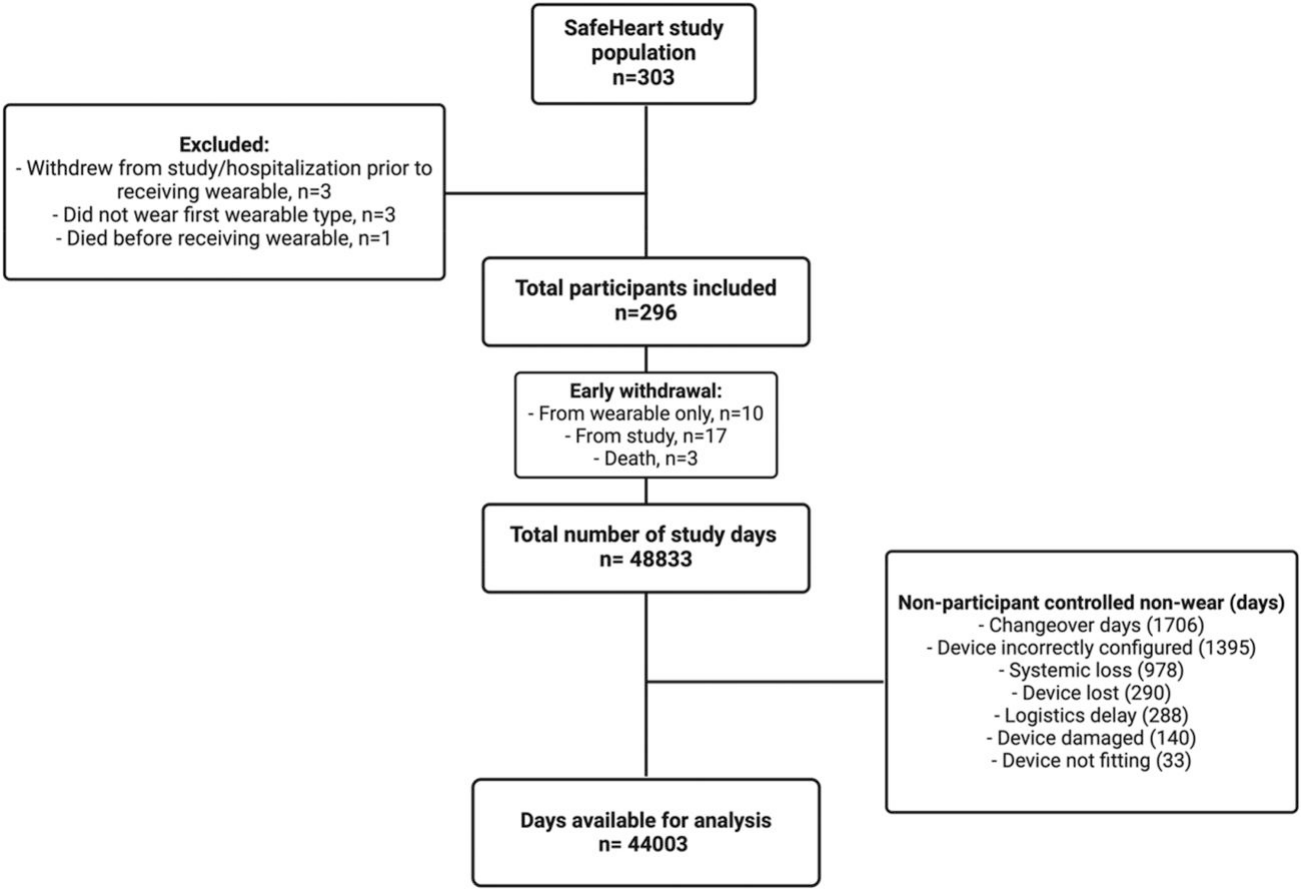
A flow chart of study participants and the number of study days. Non-participant-controlled non-wear is attributed to the operationalization of the study protocol.

A total of 303 participants were enrolled in the SafeHeart study, of which 7 participants were excluded from this study, as adherence could not be evaluated [withdrawal prior to the deployment of a wearable (*n* = 2), immediate start on another wearable used in the latter half of the SafeHeart study (*n* = 3), and death prior to the use of a wearable (*n* = 2)]. Therefore, 296 participants were included in this analysis with a total of 44 003 days in study. The median age was 64 (57–72) years, 19% were female, and 19% had a CRT-D. Baseline characteristics are presented in *Table 1*. *Figure 1* displays the total number of monitoring days, and those eligible for the calculation of adherence.

**Table 1:** Baseline characteristics among low, moderate, and optimal long-term adherence and daily adherence groups

		Long-term adherence				Daily adherence			
	Overall, *n* = 296^a^	Optimal, *n* = 83^a^	Moderate, *n* = 127^a^	Low, *n* = 86^a^	*P*-value^b^	Optimal, *n* = 163^a^	Moderate, *n* = 87^a^	Low, *n* = 46^a^	*P*-value^b^
Age (years)	64 (57–71)	65 (59–73)	64 (57–71)	62 (51–70)	0.022	65 (59–72)	62 (56–72)	60 (51–69)	0.034
Female sex	55 (19%)	16 (19%)	21 (17%)	18 (21%)	0.70	30 (18%)	15 (17%)	10 (22%)	0.80
BMI	27.6 (24.7–30.4)	26.5 (24.9–30.4)	27.6 (24.5–30.0)	28.1 (25.1–30.9)	0.50	27.4 (24.8–30.5)	27.3 (24.4–29.4)	29.0 (25.9–31.3)	0.20
Device type					>0.90				0.80
ICD	241 (81%)	67 (81%)	103 (81%)	71 (83%)		135 (83%)	69 (79%)	37 (80%)	
CRT-D	55 (19%)	16 (19%)	24 (19%)	15 (17%)		28 (17%)	18 (21%)	9 (20%)	
Years since first implantation	3 (2–5)	4 (2–9)	3 (2–5)	3 (2–5)	0.12	3 (2–5)	3 (2–6)	2 (1–4)	0.14
Device replacement	71 (24%)	22 (27%)	32 (25%)	17 (20%)	0.50	38 (23%)	25 (29%)	8 (17%)	0.30
NYHA					0.70				0.40
I	13 (19%)	3 (14%)	5 (17%)	5 (31%)		7 (18%)	3 (14%)	3 (50%)	
II	43 (63%)	15 (68%)	20 (67%)	8 (50%)		26 (65%)	15 (68%)	2 (33%)	
III	12 (18%)	4 (18%)	5 (17%)	3 (19%)		7 (18%)	4 (18%)	1 (17%)	
Myocardial infarction	108 (36%)	34 (41%)	47 (37%)	27 (31%)	0.40	66 (40%)	29 (33%)	13 (28%)	0.20
PCI	98 (33%)	27 (33%)	46 (36%)	25 (29%)	0.50	58 (36%)	27 (31%)	13 (28%)	0.60
CABG	54 (18%)	19 (23%)	24 (19%)	11 (13%)	0.20	34 (21%)	15 (17%)	5 (11%)	0.30
Previous OHCA	148 (50%)	45 (54%)	51 (40%)	52 (60%)	0.010	80 (49%)	40 (46%)	28 (61%)	0.20
HF diagnosis	161 (54%)	50 (60%)	69 (54%)	42 (49%)	0.30	89 (55%)	52 (60%)	20 (43%)	0.20
Previous HF hospitalization	22 (7.4%)	5 (6.0%)	9 (7.1%)	8 (9.3%)	0.70	8 (4.9%)	8 (9.2%)	6 (13%)	0.13
Known atrial fibrillation	104 (35%)	32 (39%)	39 (31%)	33 (38%)	0.400	60 (37%)	29 (33%)	15 (33%)	0.80
Cardiovascular comorbidity^c^	208 (70%)	63 (76%)	82 (65%)	63 (73%)	0.20	115 (71%)	64 (74%)	29 (63%)	0.40
Smoking status					0.018				0.40
Never smoked	103 (41%)	32 (43%)	35 (35%)	36 (47%)		56 (40%)	27 (39%)	20 (49%)	
Active smoker	35 (14%)	5 (6.7%)	14 (14%)	16 (21%)		16 (11%)	12 (17%)	7 (17%)	
Previous smoker	114 (45%)	38 (51%)	52 (51%)	24 (32%)		69 (49%)	31 (44%)	14 (34%)	
Medications, yes (%)									
ACE inhibitor	119 (40%)	30 (36%)	53 (42%)	36 (42%)	0.70	66 (40%)	37 (43%)	16 (35%)	0.70
ARB	72 (24%)	25 (30%)	31 (24%)	16 (19%)	0.20	43 (26%)	18 (21%)	11 (24%)	0.60
Loop diuretics	99 (33%)	31 (37%)	39 (31%)	29 (34%)	0.60	57 (35%)	29 (33%)	13 (28%)	0.70
Beta-blocker	237 (80%)	70 (84%)	101 (80%)	66 (77%)	0.50	136 (83%)	63 (72%)	38 (83%)	0.10
Calcium channel blocker	48 (16%)	15 (18%)	20 (16%)	13 (15%)	0.90	25 (15%)	14 (16%)	9 (20%)	0.80
AAD class III	48 (16%)	16 (19%)	19 (15%)	13 (15%)	0.70	27 (17%)	12 (14%)	9 (20%)	0.70
Nitrates	42 (14%)	15 (18%)	15 (12%)	12 (14%)	0.40	25 (15%)	9 (10%)	8 (17%)	0.40
ASA	102 (34%)	35 (42%)	44 (35%)	23 (27%)	0.11	62 (38%)	28 (32%)	12 (26%)	0.30
NOAC	76 (26%)	22 (27%)	30 (24%)	24 (28%)	0.80	42 (26%)	21 (24%)	13 (28%)	0.90
Warfarin	40 (14%)	11 (13%)	17 (13%)	12 (14%)	>0.90	20 (12%)	17 (20%)	3 (6.5%)	0.089
Lipid-lowering drugs	190 (64%)	57 (69%)	82 (65%)	51 (59%)	0.40	109 (67%)	56 (64%)	25 (54%)	0.30

AAD, antiarrhythmic drug; ACE inhibitor, angiotensin-converting enzyme inhibitors; AF, atrial fibrillation; ARB, angiotensin receptor blockers; ASA, acetylsalicylic acid; BMI, body mass index; CABG, coronary artery bypass graft surgery; CRT-D, implantable cardioverter defibrillator with cardiac resynchronization therapy; HF, heart failure; ICD, implantable cardioverter defibrillator; LVEF, left ventricular ejection fraction; NOAC, non-vitamin K antagonist oral anticoagulant; NYHA, New York Heart Association Functional Class; OHCA, out-of-hospital cardiac arrest; PCI, percutaneous coronary intervention; PP, primary prevention; SP, secondary prevention.

^a^Median (IQR); *n* (%).

^b^Kruskal–Wallis rank-sum test; Pearson’s χ^2^ test; Fisher’s exact test.

^c^Cardiovascular comorbidities include hypertension, hyperlipidaemia, diabetes, renal disease, chronic obstructive pulmonary disease, and obstructive sleep apnoea.

**Figure 2:**
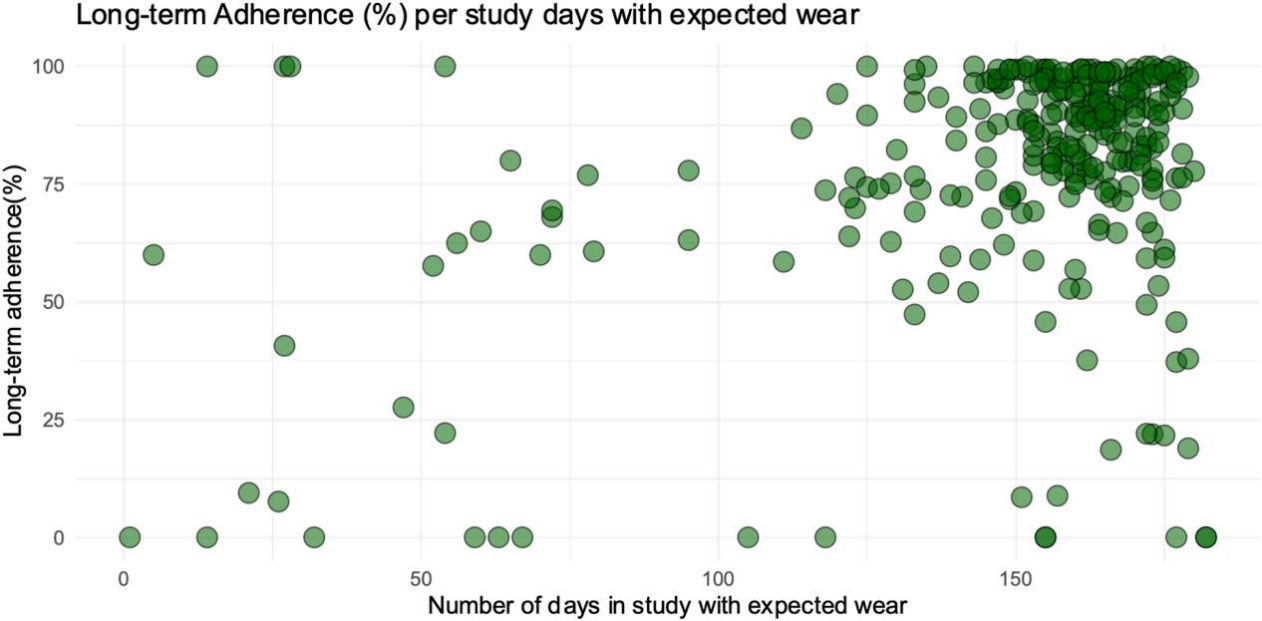
Long-term adherence (%) per participant by number of study days with expected wear. For interpretability: the dots on the far-left side on the *x*-axis represent early withdrawal from wearable use or total study withdrawal (*n* = 27).

The median long-term adherence was 88.2% (IQR 74.6–96.5), and the median number of days in the study was 160 (147–168; *Figure 2*). A comparison between the two study centres showed a statistically significant but numerically small difference in long-term adherence (86.3 vs. 88.6%, *P* = 0.04). The major reason for the decrease in long-term adherence was participant-controlled non-wear, which is presented in Supplementary material online, *Figure S3*. A total of 83 (28%), 127 (42.9%), and 86 (29.1%) participants had optimal, moderate, and low long-term adherences, respectively.

**Figure 3:**
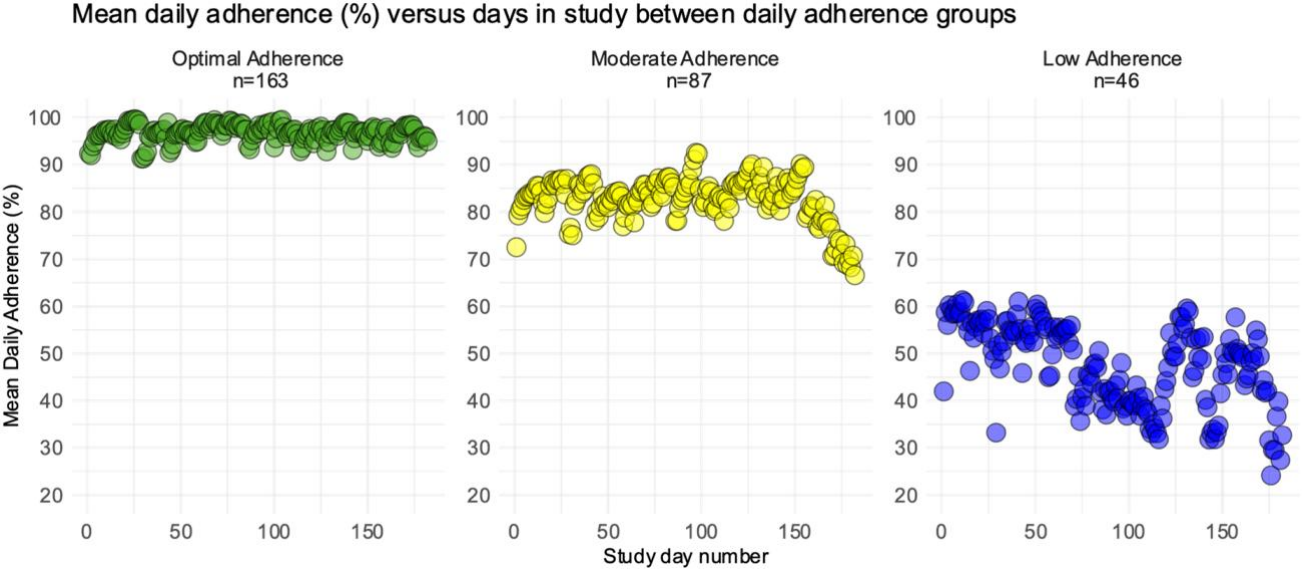
Daily adherence means throughout the study, divided by adherence groups: optimal daily adherence (90% or more), moderate adherence (75–89%), and low adherence (<75%). Analysis of variance *P* < 0.001 among groups.

The median daily adherence was 99.6% (IQR 92.0–99.8%). A comparison between the two study centres showed no difference in daily adherence (99.6% for both). Daily optimal, moderate, and low adherences were seen in 163 (55.1%), 87 (29.4%), and 46 (15.5%) participants, respectively. As depicted in *Figure 3*, there was a decline in mean daily adherence on individual study days among the optimal, moderate, and low daily adherence groups (*P* < 0.001).

### Patterns of adherence

With regard to the adherence group patterns at time points 2, 4, and 6 months, the most common combination was to remain at moderate long-term adherence throughout the whole 6 months of follow-up. A larger percentage of movements among long-term adherence groups were seen from 2 to 4 months, compared with the later time points. Dropping out was mainly seen among low adherers. All adherence combinations are given in Supplementary material online, *Figure S4* and *Table S2*.

### Data loss

**Figure 4:**
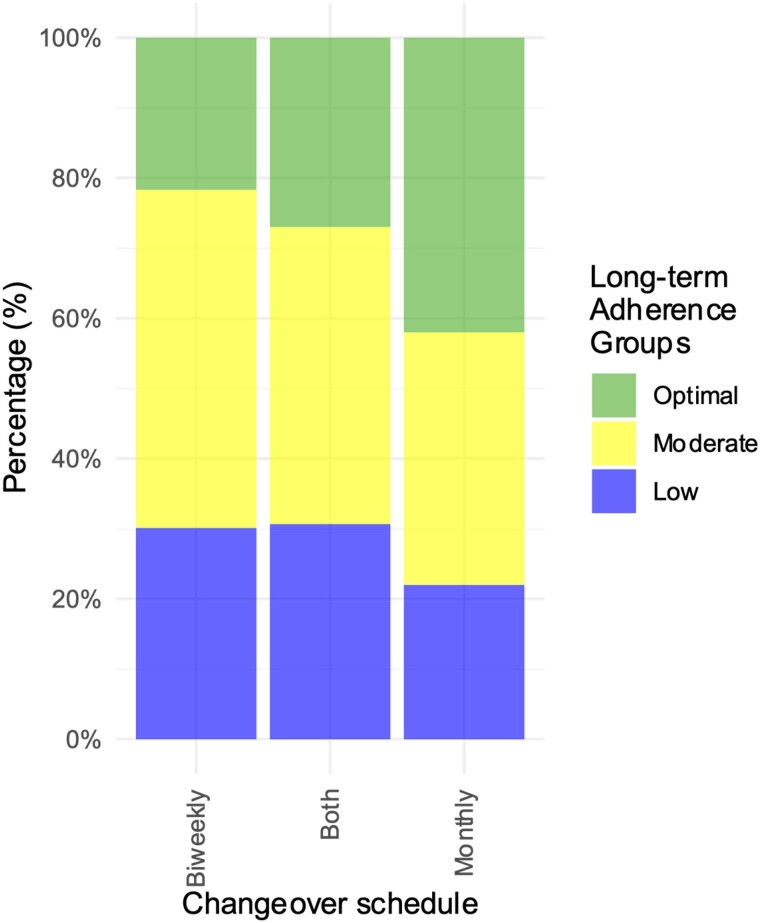
Distribution of long-term adherence between changeover schedules among the three long-term adherence groups: optimal adherence (95% or more), moderate adherence (75–94%), and low adherence (<75%). Participants were either on strictly biweekly or on monthly changeovers, or a combination of the two (i.e. both changeover schedules).

The set-up of the study (non-participant-controlled) and participant-controlled non-wear yielded 35 329 days of valid data out of the theoretical maximum of 53 872 study days, equivalent to a median data return of 74.2% (IQR 55.9–83.0). The reasons for data loss are presented in Supplementary material online, *Figure S3*. The effects of the amendment from biweekly to monthly changeovers of the wearable activity tracker are seen in *Figure 4*, where optimal adherence increased with increasing changeover interval (*P* < 0.001).

### Factors associated with adherence

A comparison of long-term and daily adherence among optimal, moderate, and low adherence groups is given in *Table 1*. For long-term adherence, age was significantly different among the groups, with participants in the low-compliance group being the youngest. Furthermore, there were more active smokers and fewer who had quit smoking in the low-adherence group. Previous out-of-hospital cardiac arrest was also more common in low adherers compared with optimal long-term adherers, but least common in moderate adherers. Type of implanted device, other cardiac diagnoses, or comorbidities, as well as medication use, were not significantly different among groups for long-term adherence. With regard to daily adherence, only age was statistically significantly different among adherence groups.

Sensitivity analyses did not show any difference in the above-described patterns for long-term adherence when splitting the population into only 2 groups, those with an adherence of 75% or more and those below. For daily adherence, age showed the same pattern, and furthermore, adherers with a daily adherence of 75% or more were typically implanted earlier than those with low adherence. All sensitivity results are summarized in Supplementary material online, *Table S3*. Baseline PROMs showed no statistically significant difference among adherence groups for either long-term or daily adherence, displayed in Supplementary material online, *Table S4*, with no change in the results in the sensitivity analyses, presented in Supplementary material online, *Table S5*.

Multivariate linear regression displayed a positive association between age and increase in the percentage of long-term adherence. Furthermore, a decreasing number of changeovers of the wearable activity tracker was associated with higher adherence. Monthly changeovers led to a relative increase in long-term adherence of 24% (*P* = 0.0047) compared with only biweekly changeovers. All linear regression results are presented in *Table 2*. Lastly, in terms of daily adherence split by calendar month, March, August, and September were the months with the lowest daily adherence, presented in Supplementary material online, *Figure S5*.

**Table 2:** Coefficient estimates for factors associated with long-term adherence from a linear regression model

Independent variables	*β* (95% CI)^a^	*P*-value
Age per 10-year increment	1.14 (1.08–1.20)	<0.001^b^
Female sex (ref.: male)	0.96 (0.85–1.09)	0.52
HF diagnosis (ref.: no)	0.98 (0.89–1.09)	0.77
Atrial fibrillation (ref.: no)	0.95 (0.85–1.06)	0.37
Cardiovascular comorbidities^b^ (ref.: no)	0.93 (0.83–1.04)	0.18
CRT-D (ref.: ICD)	1.01 (0.88–1.16)	0.88
Active smoking status (ref.: never smoked)	0.90 (0.77–1.05)	0.17
Previous smoking status (ref.: never smoked)	0.95 (0.86–1.05)	0.34
KCCQ clinical score	1.0 (0.997 -1.003)	0.80
Both changeover schedules (ref.: biweekly)	1.09 (0.97–1.22)	0.14
Monthly changeover schedules (ref.: biweekly)	1.24 (1.07–1.45)	0.0047^b^

CI, confidence interval; CRT-D, Cardiovascular resynchronization therapy defibrillator; HF, heart failure; ICD, implantable cardioverter defibrillator; KCCQ, Kansas City Cardiomyopathy Questionnaire.

^a^The dependent variable, long-term adherence, was employed as a log-transformed value in the linear regression; therefore, the coefficient estimate above reflects the relative increase in long-term adherence.

^b^Cardiovascular comorbidities include hypertension, hyperlipidaemia, diabetes, renal disease, chronic obstructive pulmonary disease, and obstructive sleep apnoea.

## Discussion

The clinical usefulness of wearable devices depends on the adherence over time. In this study, we report findings from a decentralized, international study where participants wore the activity tracker for 6 months in a row without receiving any input from the device. The high granularity of this data makes it possible to better define and understand adherence to wearables used in the clinic.

The key findings were that adherence remained consistently high over a 6-month monitoring period, with a long-term adherence of 88.2%. Older age and fewer changeover intervals of wearable devices were associated with increased adherence.

### Variables associated with adherence

The strength of our study is both a long follow-up of 6 months and continuous, rather than intermittently collected data. Previous studies of wearables and adherence predominantly looked at non-continuous data over shorter follow-up periods, most often for 7–14 days.^9,10,26^ In a study with 175 older adults, adherence to a Fitbit was high at 89% over a 30 day period. Here, the valid-day criterion was >100 steps/day. The factors associated with adherence were female sex and memory function.^27^ Most individuals carrying an ICD are older, raising the possibility of cognitive function influencing adherence, especially if the wearable activity tracker requires charging during use. However, our study did not observe this trend, possibly attributable to the choice of a research-graded wearable activity tracker that did not necessitate user charging; moreover, the median participant age was only 64 years.

The length of study participation is a factor associated with adherence, but generalizability remains difficult due to the heterogeneity of valid-day criteria. A community-based study of 711 subjects showed that only 50% still actively used their Fitbit at 6 months after purchase. The valid-day criterion was wearable and computer- or app-synced days.^13^ In ill patients, a small study of 75 participants with osteoarthritis showed a decline in adherence during the 12-week study period with 88.2% overall adherence.^28^ In a cancer chemotherapy cohort studied over a 9-month period, adherence was poor with a mean number of valid days of 44.5%, affected by a lack of prompts to encourage use.^14^ These studies defined valid days as >1500 steps/day and >10 h of use, respectively.^14,28^

With two participating sites in this study, we were presented with the possibility of comparing their adherence levels. The difference in long-term adherence was statistically significant but with a numerical difference of only 2.3% higher long-term adherence in the Danish participants compared with the Dutch participants. This comparison was done in order to examine whether adherence differed across different countries and clinics. The small difference observed may be due to variances in instructions to users, health perception and attitudes, or societal acceptance of wearable technology. Furthermore, because wearables were sent from a third country outside of the EU (UK), logistics and customs discrepancies between the two countries cannot be excluded to indirectly affect adherence. Overall, we propose that the observed increase in adherence from 86.3 to 88.6% over 6 months of follow-up may be of limited clinical relevance.

Our participants did not receive either input from the device alone or separate prompts to remind them of their use, other than indirect indicators at changeovers. The selection of an input-free device was an active choice of study design, as the purpose was to observe patients in their everyday life, without concurrent activity modification. Despite a purposedly observational aim of this study, it cannot be denied that the Hawthorne effect affects behaviour to some extent, due to participant knowledge of their being part of a study and being observed.^29^ Activity input from the device alone or coupled with encouraging prompts in the form of text messages or phone calls has been shown to improve activity.^27^ These results could likely be extrapolated to adherence.^14^

In cardiology, the main use of wearable activity trackers is undoubtedly found in either cardiovascular rehabilitation^27^ or AF detection and stroke prevention by the use of, e.g. intermittent handheld ECGs, a Fitbit or an Apple Watch.^7,10,30^ Our findings of younger age and active smoking being associated with lower adherence are consistent with studies with a shorter period of monitoring.^31–33^ In studies of intermittent handheld ECGs, medications, comorbidities, and unfavourable sociodemographic factors were found to be significantly different among those who took part in screening and those who did not. They also had worse outcomes with regard to ischaemic stroke.^10^ Older age and paroxysmal AF have been shown to be associated with higher adherence,^19^ which is in line with our findings. Consequently, considering that increasing age is often associated with more comorbidities, it could be a sign of adherence increasing with illness until a certain point, where it again declines.

### Thresholds for adherence

In the field of wearable activity trackers, it quickly becomes evident that there is a lack of consensus on cut points to define adherence levels. Prior research on wearable activity trackers predominantly used commercially available models rather than research-grade devices.^2,7,34^ These studies focus on enhancing adherence to physical activity interventions, often relying on feedback provided by the wearable or its associated mobile application. Their cut point for a valid day is often set to 10 h, depending on whether the wearable is used while the person is awake or active or based on a certain threshold for step count,^13,28,35,36^ but it may be based on the distribution of data of each individual study.^5^ To enhance generalizability and comparability, it has been suggested that the definition of adherence should imply continuous variables such as duration of wear time and number of days with valid measurements.^5^

In our study, we aimed to cover most hours of the night and day, by setting the threshold of 22 h or more to define a valid wear day. Our categorical cut points are therefore derived from nearly round-the-clock continuous wear data, ensuring a comprehensive basis for analysis. If we had allowed valid days to contain less hours of wearable data, we would have gained additional days for the analysis, while increasingly raising questions about what occurred during the non-wear period.

Lastly, when using categorical adherence levels, what characterizes someone with low adherence in continuous monitoring is a lower number of hours of wear, consequently with a larger window of no data and thereby unknown behaviours. This means that we can relatively confidently speak about trends and associations in the higher groups of adherences, whereas for low adherers, the certainty of behaviour in the hours of missing data decreases together with the decreasing wear time.

### Patterns of adherence

It is valuable to know whether, and when, a decrease in adherence over time takes place, and for how long optimal adherence is upheld. Therefore, understanding the patterns of patient motivation, engagement, and adherence to wearables is pivotal to improving the usability of digital data collection.^26^ Our findings indicate that optimal adherers are likely to maintain their adherence level throughout the entire study, with none transitioning to low long-term adherence at 4 months. Contrarily, low adherers typically begin at lower daily adherence and show a further decline within just a few weeks of participation and most often drop out. This might suggest that supportive interventions, e.g. mobile app reminders, used at a very early stage, and regularly throughout the period of study participation, could potentially improve adherence noticeably in this group. Furthermore, too frequent patient engagements may weaken long-term adherence, as testified by the fact that in this study, we saw an increase in adherence with longer changeover intervals, which acted as the only reminder about wearable use.

What is important to note is a potential difference in adherence when applying wearable use in a real-world setting, rather than in a controlled research environment. This study aimed to observe the normality of ICD patients, as well as their willingness and ability to use a wearable. Due to limited data on uniformly measured adherence from larger trials, our study aim is applicable to adherence to any simple wearable in any patient population. The wearable under examination did not require user-charging, as it, together with data extraction, was handled by investigators during changeovers. While charging may be expected in a real-world setting, the low involvement between participants and research professionals makes this study set-up widely representative. In the real-world ICD population, where wearables could be used for remote monitoring, factors such as older age, digital health literacy and socioeconomic status, or reimbursement strategies need to be accounted for, to reduce otherwise inevitable inequity.^4^

Data return rates for wearables are lacking in the literature but are critical for future studies to accurately perform sample size and power calculations. We found a high median data return of 74.2%. Data loss was primarily due to patient-controlled non-wear (13%) and thereafter due to changeover days, which are inherent to the study protocol (3.5%). Patient encouragement is, therefore, the action point with the greatest influence on total data return, but other areas in the direct control of study administration, such as logistics, should not be ignored.

In conclusion, this study proves that long-term and continuous data collection with wearable activity trackers, in a sick and elderly patient population, is feasible, with high adherence and data return. We introduce strict yet feasible cut point criteria to bridge an existing gap in the field and provide a more robust framework for accurate comparisons and interpretations in the future. There is undoubtedly benefit in improving the usability of digital wearable systems for research data collection as well as in remote monitoring of health conditions, to consequently improve preventive patient care.

### Limitations

First, the study demanded participant willingness to use the wearable activity tracker, which may introduce selection bias compared with a situation wherein a wearable is implemented as clinical routine. Second, during the course of the study, the changeover schedule of wearable activity trackers was changed from biweekly to monthly, which may, in itself, affect overall adherence. Third, information on educational level and/or socioeconomic status was not collected from the study participants, but these may be factors that potentially affect adherence. Fourth, without any standardization on adherence cut points, our study-specific thresholds may interfere with the generalizability of the results. Lastly, as there is high heterogeneity between wearable activity trackers with regard to specific functionalities, user requirements, comfort, battery life, etc., the generalizability of our results to clearly different wearables should be done with caution.

## Conclusions

We demonstrate high and consistent adherence of 88.2% to continuous use of a wearable device in an ICD population over 6 months of follow-up. Adherence was affected by participant characteristics (age, smoking habits) as well as study set-up (changeover interval). To ensure high adherence in future studies, striking a balance between interactions, expected patient engagement, and support, especially in the group of low adherers, is essential in the early planning phases of such studies.

## Supplementary Material

ztae055_Supplementary_Data

## Data Availability

The data underlying this article will be shared on reasonable request to the corresponding author.
