# Supplementary material for: Long-term adherence to a wearable for continuous behavioural activity measuring in the SafeHeart implantable cardioverter defibrillator population
Source: Eur Heart J Digit Health. 2024 Aug 1;5(5):622–32. doi: 10.1093/ehjdh/ztae055 (PMC11417489; doi:10.1093/ehjdh/ztae055)
Supplement: ztae055_Supplementary_Data [file ztae055_supplementary_data.docx]

**SUPPLEMENTARY**

**Table of contents**

1. **Supplementary Table S1-** Definitions of the types of days available in the study
2. **Supplementary Table S2-** Changes in long-term adherence at two-four and four-six months
3. **Supplementary Table S3-** Sensitivity analysis of baseline characteristics when splitting the cohort into two adherence groups
4. **Supplementary Table S4-** Baseline patient reported outcome measures between low, moderate and optimal long-term adherence and daily adherence
5. **Supplementary Table S5-** Sensitivity analysis of baseline patient reported outcome measures when splitting the cohort into two adherence groups
6. **Supplementary Figure S1**- The wearable accelerometer
7. **Supplementary Figure S2-** Distribution of number of participants for long-term adherence (%)
8. **Supplementary Figure S3-** Reasons for data loss
9. **Supplementary Figure S4-** Alluvial plot of patterns of long-term adherence at two, four and six months
10. **Supplementary Figure S5-** Seasonality of daily adherence split by calendar month

**Supplementary Table S1**

Definitions of the types of days available in the study

| **Types of days in the study** | **Definition** |
| --- | --- |
| **Valid day** | Days which have 22 hours or more of wear time. An additional constraint was added of at least 5,000 seconds of inactive time at final analysis to ensure that devices stuck in logistics transport did not incorrectly report active days. |
| **Non-participant (operational) controlled non-wear** | |
| **Changeover day** | Days when the participant needed to swap devices. These days are a specific type of systemic data loss inherent in the operationalisation of the study protocol where participants only be expected to change devices when they receive them and not at a particular time. |
| **Systemic loss** | These are days where a device has not been allocated and where receipt / dispatch has been delayed due to factors including changes of address, holidays and long hospital stays for participants and Christmas factory shutdown for the site dispatching devices. |
| **Device not fitting** | Days of missing data where the dispatch log indicated that the participant needed a longer strap. |
| **Device damaged** | Days of missing data where the dispatch log showed that the device was damaged in some way, e.g. broken strap, water ingress or data not readable. |
| **Device lost** | Days of missing data where devices where not received back from participants with otherwise high levels of data return. These included outbound losses reported by the logistics providers and devices that were never received back (inbound losses in national services or study site transfer). |
| **Logistics delay** | Days of missing data where the dispatch log showed that the device was delayed on the outbound route. These included failures of the logistics suppliers to collect packages, delays in delivery and customs delays. |
| **Device incorrectly configured** | Days of missing data where the device did not record correctly, ran out or battery early or data was lost in analysis or allocation. |
| **Participant-controlled non-wear** | |
| **Device not worn** | Days of missing data where the best explanation was non-wear. |
| **Partial day** | Days where wear time was less than 22 hours but greater than 50% (also with at least 5,000 seconds of inactive time). The reduced wear time may have been the result of end of recording time (both planned and unplanned) as well as non-wear. |
| **Device not returned** | Days of missing data from participants who persistently did not return devices. |
| **Participant refused delivery** | Days of missing data where the participant refused delivery (initially or completely). |

**Supplementary Table S2**

Changes in long-term adherence at two-four and four-six months

|  | **Change month two to four** | | | | | **Change month four to six** | | | |
| --- | --- | --- | --- | --- | --- | --- | --- | --- | --- |
| **Adherence group at two months** ^1^ | **Overall**, N = 296^12^ | **Increase**, N = 77^12^ | **Stable**, N = 137^12^ | **Decrease**, N = 67^12^ | **NA**, N = 15^12^ | **Increase**, N = 77^12^ | **Stable**, N = 137^12^ | **Decrease**, N = 67^12^ | **NA**, N = 15^12^ |
| *Optimal* | 43 (15%) | 0 (0%) | 15 (11%) | 24 (36%) | 4 (27%) | 0 (0%) | 15 (11%) | 24 (36%) | 4 (27%) |
| *Moderate* | 132 (45%) | 25 (32%) | 64 (47%) | 43 (64%) | 0 (0%) | 25 (32%) | 64 (47%) | 43 (64%) | 0 (0%) |
| *Low* | 121 (41%) | 52 (68%) | 58 (42%) | 0 (0%) | 11 (73%) | 52 (68%) | 58 (42%) | 0 (0%) | 11 (73%) |
| ^1^Optimal long-term adherence (95% or more), moderate long-term adherence (75-94%), low adherence (<75%). NA= participants that have withdrawn from either wearable use or from the study as a whole. | | | | | | | | | |
| ^2^n (%) | | | | | | | | | |

**Supplementary Table S3**

Sensitivity analysis of baseline characteristics when splitting the cohort into two adherence groups, i.e., high and low adherence based on the cutoff of 75% for long-term adherence and daily adherence, respectively

|  | **Long-term adherence** | | | | **Daily adherence** | | |
| --- | --- | --- | --- | --- | --- | --- | --- |
| **Characteristic**^1^ | **Overall**, N = 296^2^ | **High**, N = 210^2^ | **Low**, N = 86^2^ | **p-value**^13^ | **High**, N = 250^2^ | **Low**, N = 46^2^ | **p-value**^3^ |
| **Age (years)** | 64 (57, 71) | 64 (58, 72) | 62 (51, 70) | 0.019 | 64 (58, 72) | 60 (51, 69) | 0.019 |
| **Female sex** | 55 (19%) | 37 (18%) | 18 (21%) | 0.5 | 45 (18%) | 10 (22%) | 0.5 |
| **BMI** | 27.6 (24.7, 30.4) | 27.3 (24.6, 30.1) | 28.1 (25.1, 30.9) | 0.3 | 27.3 (24.6, 30.1) | 29.0 (25.9, 31.3) | 0.12 |
| **Device type** |  |  |  | 0.7 |  |  | 0.9 |
| *ICD* | 241 (81%) | 170 (81%) | 71 (83%) |  | 204 (82%) | 37 (80%) |  |
| *CRT-D* | 55 (19%) | 40 (19%) | 15 (17%) |  | 46 (18%) | 9 (20%) |  |
| **Years since first implantation** | 3 (2, 5) | 3 (2, 6) | 3 (2, 5) | 0.11 | 3 (2, 6) | 2 (1, 4) | 0.046 |
| **Device replacement** | 71 (24%) | 54 (26%) | 17 (20%) | 0.3 | 63 (25%) | 8 (17%) | 0.3 |
| **NYHA** |  |  |  | 0.3 |  |  | 0.11 |
| *I* | 13 (19%) | 8 (15%) | 5 (31%) |  | 10 (16%) | 3 (50%) |  |
| *II* | 43 (63%) | 35 (67%) | 8 (50%) |  | 41 (66%) | 2 (33%) |  |
| *III* | 12 (18%) | 9 (17%) | 3 (19%) |  | 11 (18%) | 1 (17%) |  |
| **Ischemic heart disease** | 153 (52%) | 113 (54%) | 40 (47%) | 0.3 | 134 (54%) | 19 (41%) | 0.13 |
| **Myocardial infarction** | 108 (36%) | 81 (39%) | 27 (31%) | 0.2 | 95 (38%) | 13 (28%) | 0.2 |
| **PCI** | 98 (33%) | 73 (35%) | 25 (29%) | 0.3 | 85 (34%) | 13 (28%) | 0.4 |
| **CABG** | 54 (18%) | 43 (20%) | 11 (13%) | 0.12 | 49 (20%) | 5 (11%) | 0.2 |
| **Previous OHCA** | 148 (50%) | 96 (46%) | 52 (60%) | 0.021 | 120 (48%) | 28 (61%) | 0.11 |
| **HF diagnosis** | 161 (54%) | 119 (57%) | 42 (49%) | 0.2 | 141 (56%) | 20 (43%) | 0.11 |
| **Previous HF hospitalization** | 22 (7.4%) | 14 (6.7%) | 8 (9.3%) | 0.4 | 16 (6.4%) | 6 (13%) | 0.13 |
| **Known atrial fibrillation** | 104 (35%) | 71 (34%) | 33 (38%) | 0.5 | 89 (36%) | 15 (33%) | 0.7 |
| **Cardiovascular comorbidity** | 208 (70%) | 145 (69%) | 63 (73%) | 0.5 | 179 (72%) | 29 (63%) | 0.2 |
| **Smoking status** |  |  |  | 0.008 |  |  | 0.3 |
| *Never smoked* | 103 (41%) | 67 (38%) | 36 (47%) |  | 83 (39%) | 20 (49%) |  |
| *Active smoker* | 35 (14%) | 19 (11%) | 16 (21%) |  | 28 (13%) | 7 (17%) |  |
| *Previous smoker* | 114 (45%) | 90 (51%) | 24 (32%) |  | 100 (47%) | 14 (34%) |  |
| **ACE inhibitor** | 119 (40%) | 83 (40%) | 36 (42%) | 0.7 | 103 (41%) | 16 (35%) | 0.4 |
| **ARB** | 72 (24%) | 56 (27%) | 16 (19%) | 0.14 | 61 (24%) | 11 (24%) | >0.9 |
| **Loop diuretics** | 99 (33%) | 70 (33%) | 29 (34%) | >0.9 | 86 (34%) | 13 (28%) | 0.4 |
| **betablocker** | 237 (80%) | 171 (81%) | 66 (77%) | 0.4 | 199 (80%) | 38 (83%) | 0.6 |
| **calcium channel blocker** | 48 (16%) | 35 (17%) | 13 (15%) | 0.7 | 39 (16%) | 9 (20%) | 0.5 |
| **AAD class III** | 48 (16%) | 35 (17%) | 13 (15%) | 0.7 | 39 (16%) | 9 (20%) | 0.5 |
| **nitrates** | 42 (14%) | 30 (14%) | 12 (14%) | >0.9 | 34 (14%) | 8 (17%) | 0.5 |
| **ASA** | 102 (34%) | 79 (38%) | 23 (27%) | 0.074 | 90 (36%) | 12 (26%) | 0.2 |
| **NOAC** | 76 (26%) | 52 (25%) | 24 (28%) | 0.6 | 63 (25%) | 13 (28%) | 0.7 |
| **warfarin** | 40 (14%) | 28 (13%) | 12 (14%) | 0.9 | 37 (15%) | 3 (6.5%) | 0.13 |
| **lipid lowering drugs** | 190 (64%) | 139 (66%) | 51 (59%) | 0.3 | 165 (66%) | 25 (54%) | 0.13 |
| ^1^AAD, antiarrhythmic drug; ACE inhibitor, Angiotensin-converting enzyme inhibitors; AF, atrial fibrillation; ARB, angiotensin receptor blockers; ASA, acetylsalicylic acid; BMI, body mass index; CABG, coronary artery bypass graft surgery; CRT-D, Implantable Cardioverter Defibrillator with cardiac resynchronization therapy; HF, heart failure; ICD, Implantable Cardioverter Defibrillator; LVEF, left ventricular ejection fraction; NOAC, non-vitamin K antagonist oral anticoagulant; NYHA, New York Heart Association Functional Class; OHCA, out-of-hospital cardiac arrest; PCI, percutaneous coronary intervention; PP, primary prevention; SP, secondary prevention. **Cardiovascular comorbidities include hypertension, hyperlipidemia, diabetes, renal disease, chronic obstructive pulmonary disease, and obstructive sleep apnea. | | | | | | | |
| ^2^Median (IQR); n (%) | | | | | | | |
| ^3^Wilcoxon rank sum test; Pearson's Chi-squared test; Fisher's exact test | | | | | | | |

**Supplementary Table S4**

Baseline patient reported outcome measures between low, moderate and optimal long-term adherence and daily adherence

|  | **Long-term adherence** | | | | | **Daily adherence** | | | |
| --- | --- | --- | --- | --- | --- | --- | --- | --- | --- |
| **Characteristic**^1^ | **Overall**, N = 296^2^ | **Optimal**, N = 83^2^ | **Moderate**, N = 127^2^ | **Low**, N = 86^2^ | **p-value**^3^ | **Optimal**, N = 163^2^ | **Moderate**, N = 87^2^ | **Low**, N = 46^2^ | **p-value**^3^ |
| EQ5D-5L^1^ |  |  |  |  |  |  |  |  |  |
| **Utility score** | 0.91 (0.81, 1.00) | 0.92 (0.84, 1.00) | 0.91 (0.81, 1.00) | 0.89 (0.75, 1.00) | 0.5 | 0.92 (0.82, 1.00) | 0.89 (0.80, 1.00) | 0.92 (0.79, 1.00) | 0.7 |
| **VAS score** | 80 (63, 90) | 80 (62, 90) | 80 (60, 90) | 79 (70, 87) | 0.7 | 78 (62, 90) | 80 (65, 90) | 80 (70, 89) | 0.9 |
| KCCQ^1^ |  |  |  |  |  |  |  |  |  |
| **Symptoms score** | 85 (71, 98) | 86 (73, 100) | 88 (70, 98) | 83 (71, 98) | 0.7 | 85 (72, 98) | 85 (71, 100) | 83 (73, 96) | >0.9 |
| **Physical limitations score** | 88 (75, 96) | 88 (71, 96) | 88 (75, 96) | 92 (75, 100) | 0.7 | 88 (71, 96) | 88 (75, 96) | 92 (75, 100) | 0.7 |
| **Social limitations score** | 88 (63, 100) | 88 (63, 100) | 85 (63, 100) | 85 (57, 100) | >0.9 | 88 (63, 100) | 83 (63, 100) | 92 (63, 100) | 0.6 |
| **QoL score** | 83 (67, 92) | 83 (67, 100) | 83 (67, 92) | 75 (58, 98) | 0.6 | 83 (67, 92) | 83 (58, 92) | 75 (67, 100) | >0.9 |
| **Self-efficacy score** | 75 (63, 100) | 88 (75, 100) | 75 (63, 88) | 75 (63, 88) | 0.3 | 75 (75, 100) | 75 (63, 88) | 88 (63, 88) | 0.7 |
| **Clinical score** | 86 (73, 96) | 85 (71, 97) | 88 (73, 96) | 84 (75, 96) | >0.9 | 86 (72, 96) | 88 (73, 96) | 84 (76, 97) | >0.9 |
| **Summary score** | 85 (69, 95) | 83 (72, 96) | 87 (69, 94) | 84 (68, 96) | >0.9 | 84 (70, 95) | 86 (69, 94) | 85 (70, 97) | 0.9 |
| ^1^EQ5D-5L, EuroQol-5 Dimension self-assessed health-related quality of life questionnaire; KCCQ, Kansas City Cardiomyopathy Questionnaire; VAS, Visual Analogue Scale; QoL, Quality of life | | | | | | | | | |
| ^2^Median (IQR) | | | | | | | | | |
| ^3^Kruskal-Wallis rank sum test | | | | | | | | | |

| **Supplementary Table S5** |
| --- |

Sensitivity analysis of baseline patient reported outcome measures when splitting the cohort into two adherence groups, i.e., high and low adherence based on the cutoff of 75% for long-term adherence and daily adherence, respectively

|  | **Long-term adherence** | | | | **Daily adherence** | | |
| --- | --- | --- | --- | --- | --- | --- | --- |
| **Characteristic**^1^ | **Overall**, N = 296^2^ | **High**, N = 210^2^ | **Low**, N = 86^2^ | **p-value**^3^ | **High**, N = 250^2^ | **Low**, N = 46^2^ | **p-value**^3^ |
| **Utility score** | 0.91 (0.81, 1.00) | 0.92 (0.82, 1.00) | 0.89 (0.75, 1.00) | 0.3 | 0.91 (0.81, 1.00) | 0.92 (0.79, 1.00) | 0.7 |
| **VAS score** | 80 (63, 90) | 80 (61, 90) | 79 (70, 87) | 0.8 | 80 (63, 90) | 80 (70, 89) | 0.7 |
| **Symptoms score** | 85 (71, 98) | 88 (71, 100) | 83 (71, 98) | 0.4 | 85 (71, 98) | 83 (73, 96) | 0.7 |
| **Physical limitations score** | 88 (75, 96) | 88 (71, 96) | 92 (75, 100) | 0.5 | 88 (74, 96) | 92 (75, 100) | 0.5 |
| **Social limitations score** | 88 (63, 100) | 88 (63, 100) | 85 (57, 100) | 0.7 | 88 (63, 100) | 92 (63, 100) | 0.4 |
| **QoL score** | 83 (67, 92) | 83 (67, 92) | 75 (58, 98) | 0.3 | 83 (65, 92) | 75 (67, 100) | 0.8 |
| **Self-efficacy score** | 75 (63, 100) | 75 (75, 100) | 75 (63, 88) | 0.4 | 75 (63, 100) | 88 (63, 88) | >0.9 |
| **Clinical score** | 86 (73, 96) | 88 (72, 96) | 84 (75, 96) | >0.9 | 87 (72, 96) | 84 (76, 97) | >0.9 |
| **Summary score** | 85 (69, 95) | 85 (70, 95) | 84 (68, 96) | >0.9 | 85 (69, 95) | 85 (70, 97) | 0.7 |
| ^1^EQ5D-5L, EuroQol-5 Dimension self-assessed health-related quality of life questionnaire; KCCQ, Kansas City Cardiomyopathy Questionnaire; VAS, Visual Analogue Scale; QoL, Quality of life | | | | | | | |
| ^2^Median (IQR) | | | | | | | |
| ^3^Wilcoxon rank sum test | | | | | | | |

**Supplementary Figure S1**

**
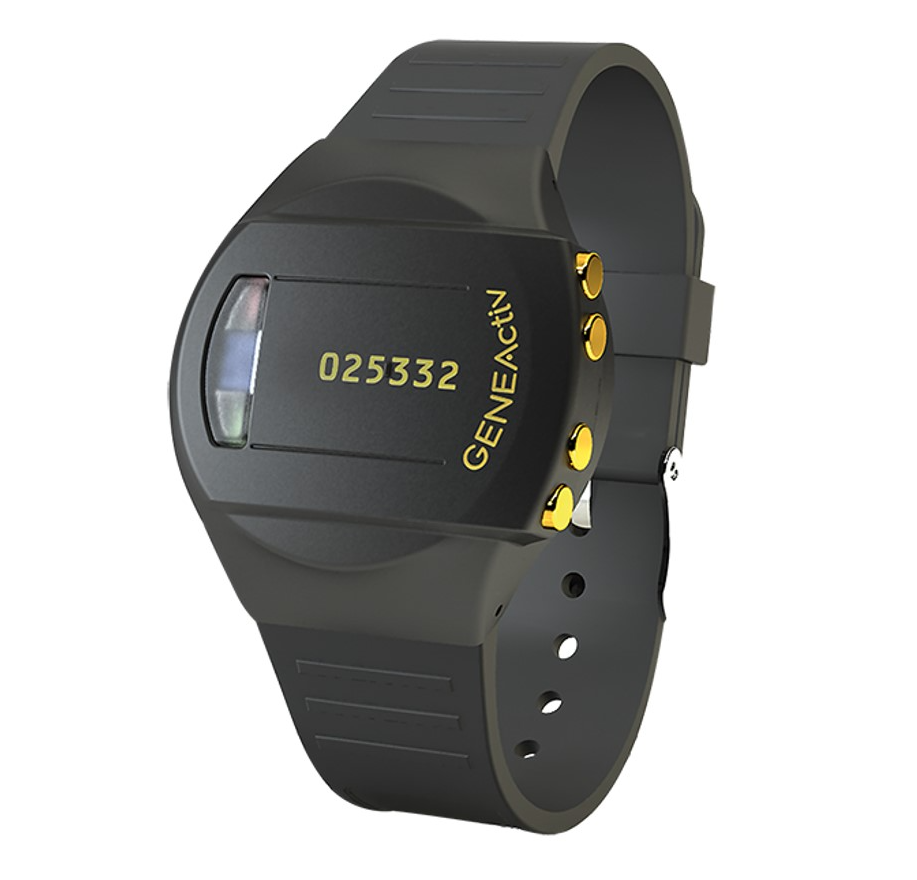
**

Caption: The wearable used in the study, the GENEActiv Original 1.1 triaxial accelerometer (Activinsights Ltd, Cambridgeshire, United Kingdom) **Supplementary Figure S2**

**
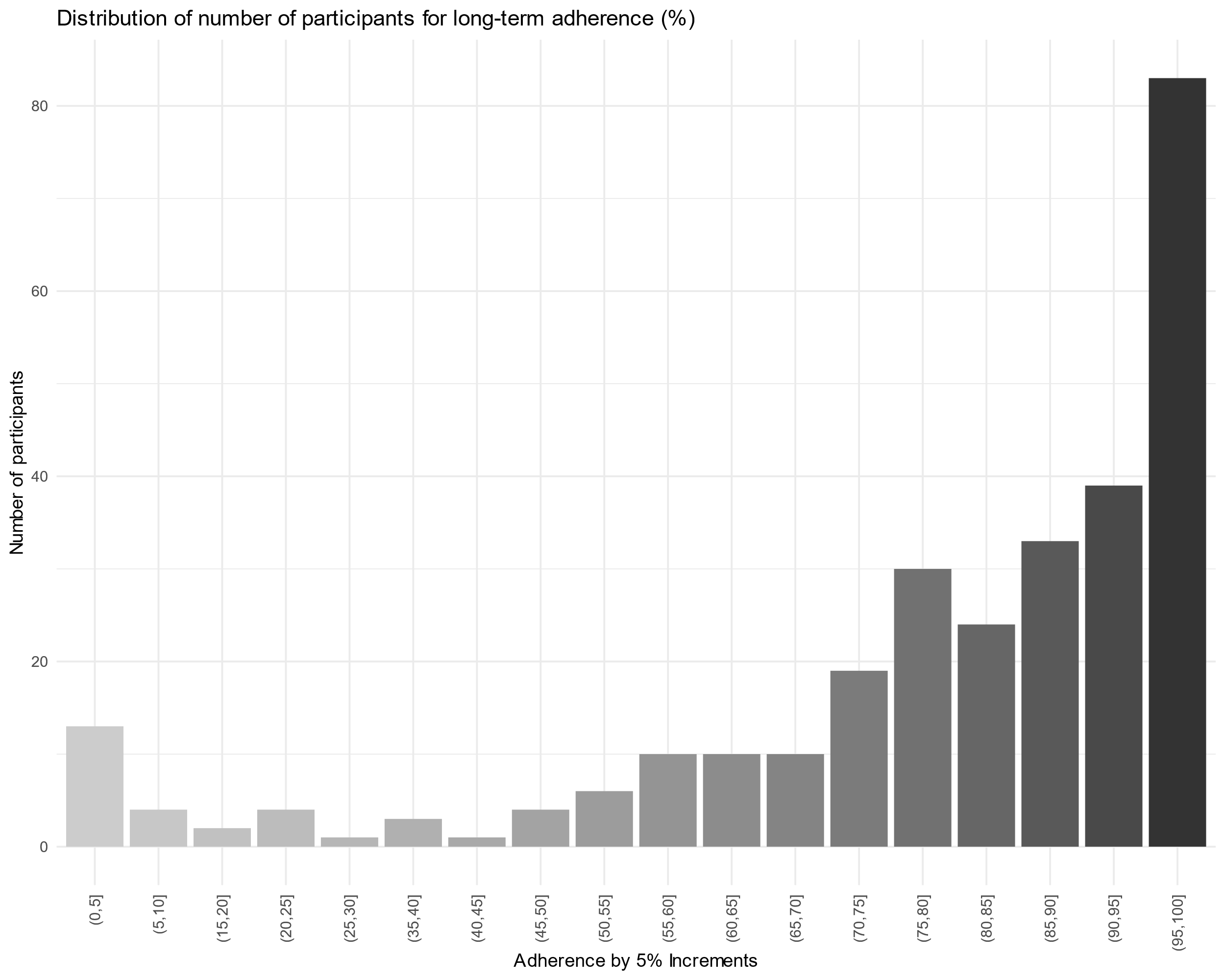
**

Caption: Long-term adherence (%) for each participant, grouped into 5% increments.

**Supplementary Figure S3**


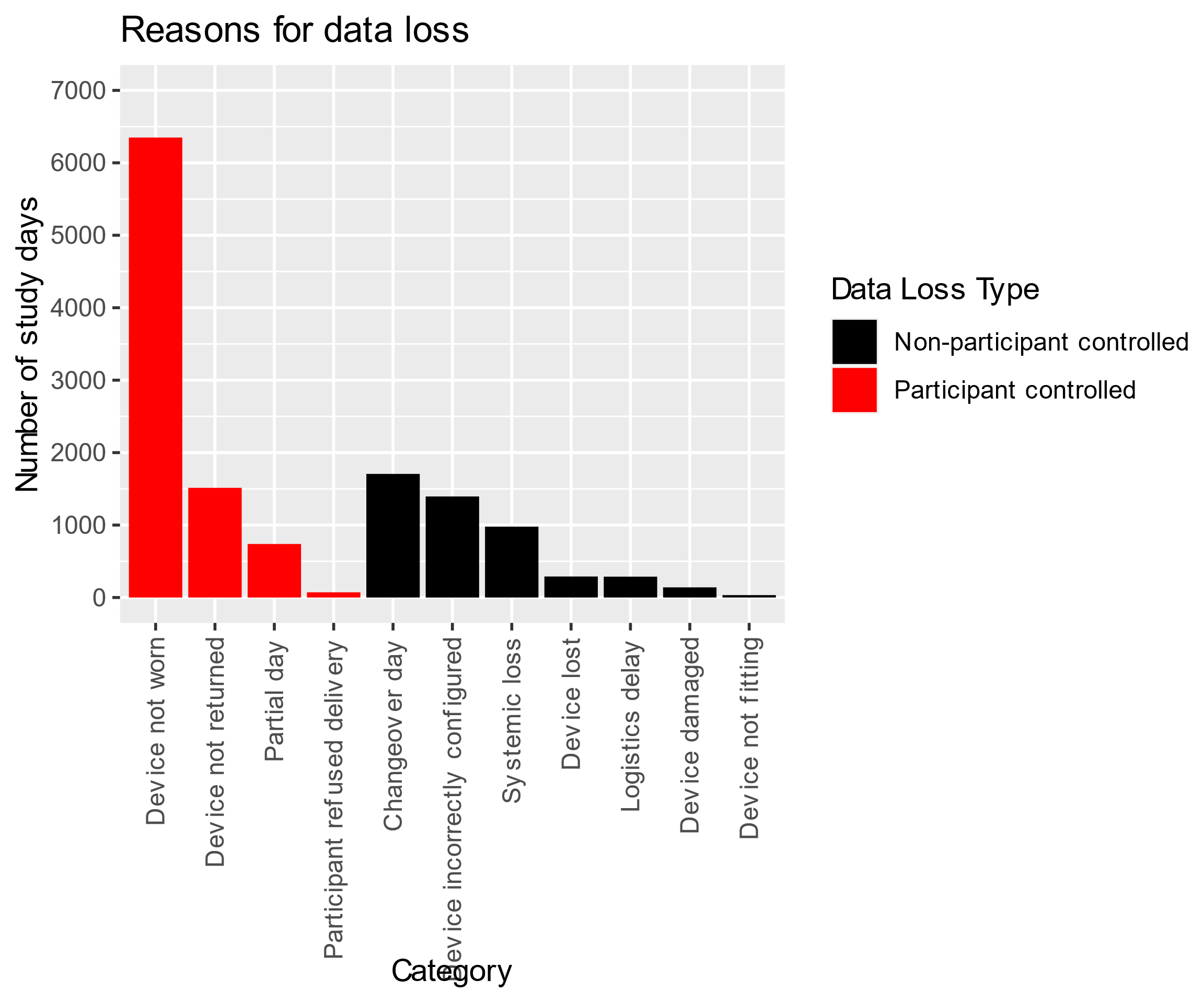


Caption: Reasons for data loss, divided into categories and overall type of data loss. Total number of valid days was 35,329 days, after the above days were deducted. Participant controlled non-wear was where the participants could have worn the device but chose not to.

**Supplementary Figure S4**

Alluvial plot of patterns of long-term adherence at two, four and six months

**
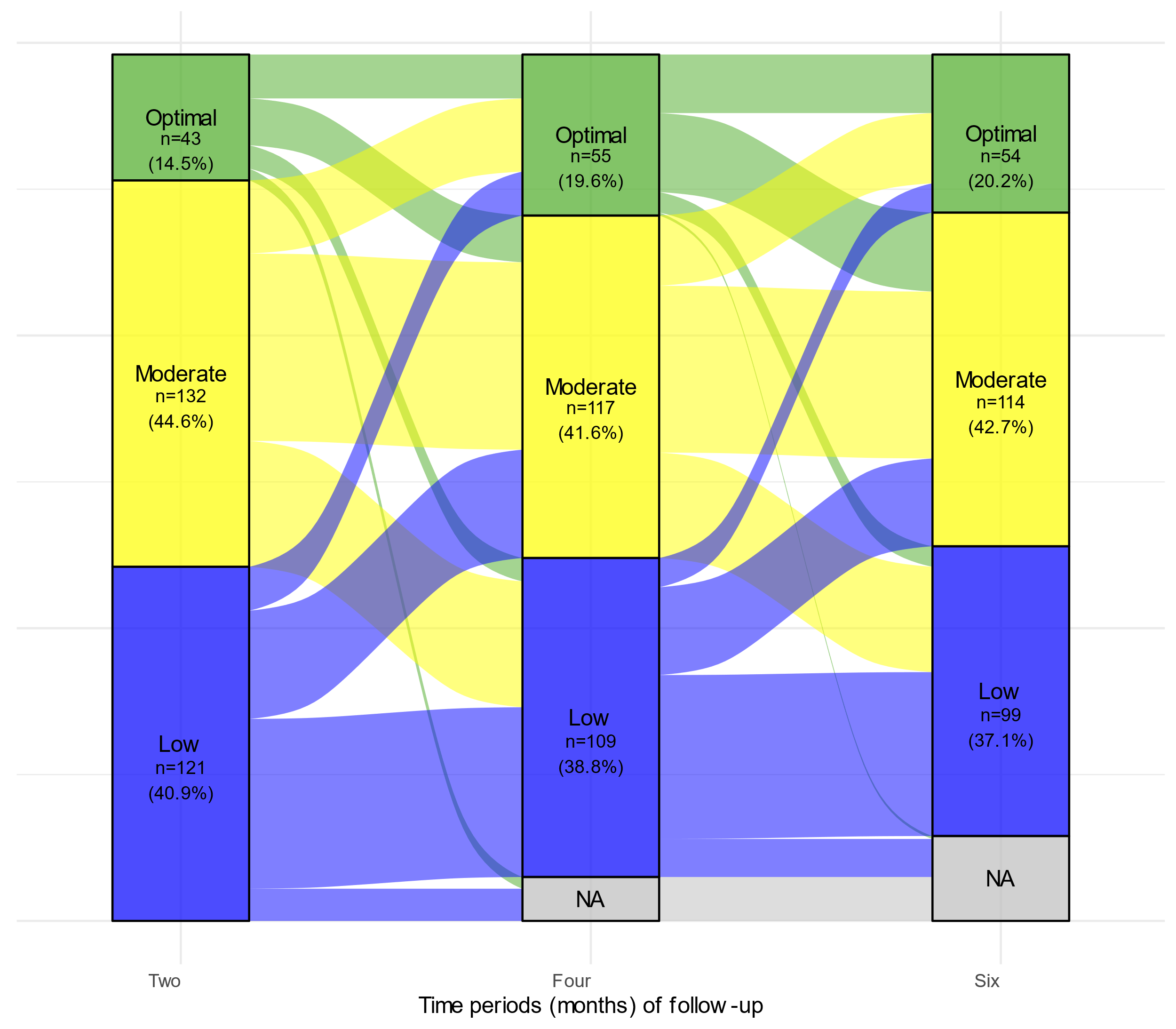
**

Caption: Patterns of long-term adherence at time points two, four and six months, total n=296. For each participant their long-term adherence group (y-axis) was defined for months 1-2(two), 3-4(four) and 5-6 (six) on the x-axis. The width of the alluvial defines the frequency of the pattern.

Optimal long-term adherence (95% or more), moderate long-term adherence (75-94%), low adherence (<75%).

NA = Participants that have withdrawn from either wearable use or from the study as a whole by the time of two, four and six months.

**Supplementary Figure S5**

Seasonality of daily adherence split by calendar month

**
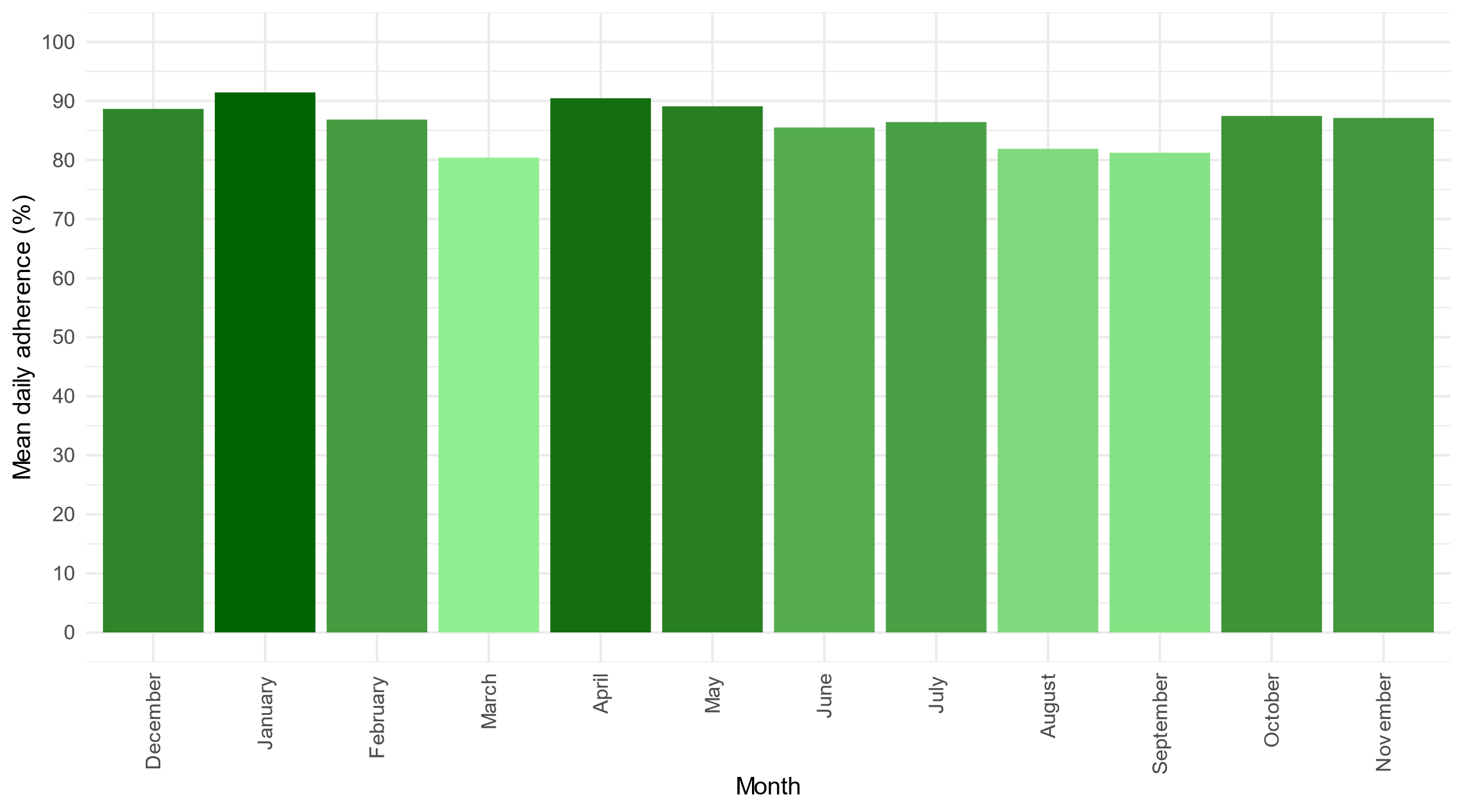
**

Caption: Seasonality trend for mean daily adherence per calendar months December 2021- November 2022. Each participant follow up is maximum 6 months. Only study days of potential wear have been included. Data is not normally distributed, where the medians per month were 99.53-99.81%.
